# Immunotherapy of Liver Diseases

**DOI:** 10.1155/2019/9343505

**Published:** 2019-08-21

**Authors:** Dechun Feng, YinYing Lu, Xiaoni Kong, Feng Li

**Affiliations:** ^1^Laboratory of Liver Diseases, National Institute on Alcohol Abuse and Alcoholism, National Institutes of Health, Bethesda, MD, USA; ^2^Comprehensive Liver Cancer Center, Beijing 302 Hospital, Beijing, China; ^3^Department of Liver Surgery, Renji Hospital, School of Medicine, Shanghai Jiao Tong University, Shanghai, China; ^4^Department of Molecular and Cellular Biology, Baylor College of Medicine, Houston, TX, USA

As an immunological organ, the liver played critical roles against invading pathogens. Many immune cells such as Kupffer cells, natural killer (NK) cells, and NKT cells were located in the liver under normal physiological condition. Under pathological condition, such as acute or chronic liver injury, more immune cells migrated into the liver. These cells may act as friends to clear damaged hepatocytes, promote liver repair, improve the resolution of liver fibrosis, and eliminate liver cancer cells. They may also play bad roles in the liver, such as further damaging hepatocytes, promoting hepatic stellate cell activation and live fibrosis, and facilitating tumor growth. So, how to understand the detailed regulation mechanisms in liver immune cells and how to target immune cells in the liver to improve the therapy of liver diseases are a challenging problem in the liver study field.

In this special issue, we have invited a few Research Articles and Reviews that address such issues.

In the paper titled “Elevated Serum IgG Levels Positively Correlated with IL-27 May Indicate Poor Outcome in Patients with HBV-Related Acute-On-Chronic Liver Failure,” the authors studied the correlation of serum immunoglobulins including IgG, IgA, and IgM and interleukin-27 (IL-27) in patients with HBV-related acute-on-chronic liver failure (HBV-ACLF). They compared HBV-ACLF patients with chronic hepatitis B (CHB) patients as well as normal control. They found that serum immunoglobulins were preferentially elevated in HBV-ACLF patients. In addition, serum IgG levels were positively correlated with IL-27. This study may be helpful for predicting prognosis in HBV-ACLF patients.

In the paper titled “Immunoregulatory Effect of Koumine on Nonalcoholic Fatty Liver Disease Rats,” the authors studied the effects of koumine, the main and active ingredient isolated from *Gelsemium elegans*, on a rat nonalcoholic fatty liver disease (NAFLD) model. They showed that Koumine could significantly reduce improved liver injury in NAFLD rats. Koumine treatment also decreased the percentages of Th1 and Th17 cells and increased Th2 and Treg cells in the liver.

In the paper titled “Overexpression of Tumor Necrosis Factor-Like Ligand 1 A in Myeloid Cells Aggravates Liver Fibrosis in Mice,” the authors studied how myeloid cell derived TNF-like ligand 1 aberrance (TL1A) contributed to the development of liver fibrosis. They found that overexpression of TL1A in myeloid cells accelerated the necrosis and apoptosis of hepatocytes and promoted activation of hepatic stellate cells (HSCs). Moreover, TL1A overexpression in macrophages promoted secretion of platelet-derived growth factor-BB (PDGF-BB), tumor necrosis factor-*α* (TNF-*α*), and interleukin-1*β* (IL-1*β*) which further activated HSCs and deteriorated liver fibrosis.

In the paper titled “Immunomodulatory Effects of Combination Therapy with Bushen Formula plus Entecavir for Chronic Hepatitis B Patients,” the authors evaluated the beneficial effects of the traditional Chinese medicine Bushen formula (BSF) in combination of plus entecavir (ETV) in naïve chronic hepatitis B (CHB) patients and that in CHB patients with partial virological response to ETV. They found that the combination therapy with BSF plus ETV increased Th1 and DC frequencies and decreased Treg frequency in naïve CHB patients. In CHB patients with partial virological response to ETV, the combination therapy downregulated PD-L1 levels on DCs and the frequency of Treg. The modulation of the immune system in these patients with BSF was related to HBsAg decline.

In the paper titled “The Crosstalk between Fat Homeostasis and Liver Regional Immunity in NAFLD,” the authors reviewed how liver nonparenchymal cell, adipocytes, and hepatocytes crosstalk with each other in the development of NAFLD and NASH. They also summarized how ncRNAs (including miRNAs and lncRNAs) participated in the pathological process of NAFLD by changing body fat homeostasis.

In the paper titled “Alda-1 Ameliorates Liver Ischemia-Reperfusion Injury by Activating Aldehyde Dehydrogenase 2 and Enhancing Autophagy in Mice,” the authors investigated the novel role of acetaldehyde metabolizing enzyme ALDH2 in ischemia-reperfusion injury (IRI). Pretreatment of ALDH2 activator Alda-1 protects mice from IRI. Detailed mechanism study revealed that Alda-1 treatment could activate AMPK and autophagy which was very helpful to remove damaged organelles and protected hepatocyte from necrosis and apoptosis. These findings collectively indicate that Alda-1-mediated ALDH2 activation could be a promising strategy to improve liver IRI by clearance of reactive aldehydes and enhancement of autophagy.

In the paper titled “Total HLA Class I Antigen Loss with the Downregulation of Antigen-Processing Machinery Components in Two Newly Established Sarcomatoid Hepatocellular Carcinoma Cell Lines,” the authors studied HLA class I antigen abnormalities in sarcomatoid hepatocellular carcinoma (sHCC). They analyzed the growth characteristics and HLA class I antigen status of four sHCC cell lines. Cell lines with nondetectable surface HLA class I antigen expression, intracellular *β*2-microglobulin (*β*2m) and marked HLA class I heavy chain, and selective antigen-processing machinery (APM) components showed enhanced growth ability. These findings may have implications for a proper design of T cell immunotherapy for the treatment of sHCC patients.

In the paper titled “Mesenchymal Stem Cells Ameliorate Hepatic Ischemia/Reperfusion Injury via Inhibition of Neutrophil Recruitment” that studied the protective effects of mesenchymal stem cells (MSCs) in a rat liver ischemia/reperfusion injury (IRI) model” the authors showed that treatment with MSCs protected rat against hepatic IRI and attenuated hepatic neutrophil infiltration. The protective effects may be attributed to the decreased expression of CXCR2 on the surface of neutrophils and reduced CXCL2 production in macrophages. MSCs can significantly ameliorate hepatic IRI predominantly through its inhibitory effect on hepatic neutrophil migration and infiltration.

In the paper titled “RIPK3-Mediated Necroptosis and Neutrophil Infiltration Are Associated with Poor Prognosis in Patients with Alcoholic Cirrhosis,” the authors explored the immunomodulatory effects of mesenchymal stem cells (MSCs) on liver IRI. They showed that treatment with MSCs protected rat against hepatic IRI, with significantly decreased liver damage and hepatic neutrophil infiltration. The mechanisms can be attributed to reduced CXCR2 expression on neutrophils and diminished CXCL2 production in macrophages.

In the paper titled “Enhanced Regeneration and Hepatoprotective Effects of Interleukin 22 Fusion Protein on a Predamaged Liver Undergoing Partial Hepatectomy,” the authors studied whether the RIPK3 level is correlated with neutrophil infiltration or poor prognosis in alcoholic cirrhotic patients. They analyzed 20 samples from alcoholic cirrhotic patients 5 normal liver samples. The results showed that the MPO and RIPK3 levels in the liver were positively related to the Ishak score. The RIPK3 was also significantly and positively related to the Knodell score. The study suggested that RIPK3-mediated necroptosis and neutrophil-mediated alcoholic liver inflammatory response are highly correlated with poor prognosis in patients with end-stage alcoholic cirrhosis.

The tenth paper discussed the beneficial role of interleukin 22 (IL-22) in liver regeneration deficiency in chronic liver disease patients and liver IRI after surgery. They found that IL-22 treatment prior to IRI effectively reduced liver damage through decreased liver injury and improved liver histology. IL-22 can also promote liver regeneration in mice with predamaged livers following PHx. IL-22 may be considered as a promising therapeutic agent to improve liver regeneration deficiency and liver IRI in patients.

In the paper titled “Complement System as a Target for Therapies to Control Liver Regeneration/Damage in Acute Liver Failure Induced by Viral Hepatitis,” the authors evaluated the role of complement components in acute liver failure (ALF) caused by viral hepatitis. They found low levels of C3a in plasma samples with high frequency of C3a, C5a, and C5b/9 deposition in liver parenchyma. The data suggested that the complement system may be involved in liver dysfunction in viral-induced acute liver failure.

In the paper titled “Natural Killer Cells in Liver Disease and Hepatocellular Carcinoma and the NK Cell-Based Immunotherapy,” the authors reviewed the NK cell phenotypic and functional changes in liver diseases. They discussed the role of NK cells in chronic viral hepatitis, alcoholic liver diseases, nonalcoholic fatty liver disease (NAFLD)/NASH, and hepatocellular carcinoma (HCC). In the review, NK cell-based immunotherapy for cancer was also discussed.

In the paper titled “Construction and Characterization of Adenovirus Vectors Encoding Aspartate-*β*-Hydroxylase to Preliminary Application in Immunotherapy of Hepatocellular Carcinoma,” the authors described a DC vaccine targeting aspartate-*β*-hydroxylase (AAH), a tumor-associated cell surface protein. They tested the antitumor effect of the vaccine in HepG2 cells and found significantly enhanced lysis effect of cytotoxic T lymphocytes (CTLs) in the vaccine group. The approach can be considered as a potential candidate for DC-based immunotherapy of HCC.

In the paper titled “The Imbalance between Foxp3+Tregs and Th1/Th17/Th22 Cells in Patients with Newly Diagnosed Autoimmune Hepatitis,” the authors studied the numbers of Foxp3+Tregs and Th1-Th17-Th22 cells newly diagnosed autoimmune hepatitis (AIH) patients. They showed that active AIH patients had significantly decreased numbers of Foxp3+Tregs and increased numbers of Th1/Th17/Th22 cells. Also, the serum levels of IL-17A and IL-22 were correlated positively with liver injury. The findings demonstrated that an imbalance between Tregs and Th1-Th17-Th22 cells might contribute to the pathogenic process of AIH.

